# Retraction Note: Novel two-stage surgical treatment for Cantrell syndrome complicated by severe pulmonary hypertension: a case report

**DOI:** 10.1186/s13256-015-0573-0

**Published:** 2015-03-26

**Authors:** Xiaochen Wu, Jinbao Zhang, Hui Ouyang, Qin Yue, Heng Zhao

**Affiliations:** 1 Department of Cardiovascular, Chengdu Millitary General Hospital, 270th Rongdu Road, Chengdu City, 610083 China; 2 Department of Ultrasound, Chengdu Millitary General Hospital, 270th Rongdu Road, Chengdu City, 610083 China

## Retraction

This article [1] has been retracted by the Editor. Although the patient’s legal guardian originally gave consent for publication of the case and accompanying images, we were informed that they withdrew this consent shortly after publication. The article is no longer available online in order to protect patient confidentiality.
